# Circ0120816 acts as an oncogene of esophageal squamous cell carcinoma by inhibiting miR-1305 and releasing TXNRD1

**DOI:** 10.1186/s12935-020-01617-w

**Published:** 2020-10-29

**Authors:** Xiaoyong Li, Laichun Song, Bo Wang, Chao Tao, Lei Shi, Ming Xu

**Affiliations:** Department of Cardiac Surgery, Hubei Province Key Laboratory of Occupational Hazard Identification and Control, Wuhan Asia Heart Hospital Affiliated to Wuhan University of Science and Technology, No.753 Jinghan Road, Wuhan, 430022 Hubei China

**Keywords:** circ0120816, miR-1305, TXNRD1, Esophageal squamous cell carcinoma

## Abstract

**Background:**

Circular RNAs (circRNAs) have been discovered to participate in the carcinogenesis of multiple cancers. However, the role of circRNAs in esophageal squamous cell carcinoma (ESCC) progression is yet to be properly understood. This research aimed to investigate and understand the mechanism used by circRNAs to regulate ESCC progression.

**Methods:**

Bioinformatics analysis was first performed to screen dysregulated circRNAs and differentially expressed genes in ESCC. The ESCC tissue samples and adjacent normal tissue samples utilized in this study were obtained from 36 ESCC patients. All the samples were subjected to qRT-PCR analysis to identify the expression of TXNRD1, circRNAs, and miR-1305. Luciferase reporter assay, RNA immunoprecipitation assay and RNA pull-down assay were later conducted to verify the existing relationship among circ0120816, miR-1305 and TXNRD1. CCK-8, BrdU, cell adhesion, cell cycle, western blot and caspase 3 activity assays were also employed to evaluate the regulation of these three biological molecules in ESCC carcinogenesis. To evaluate the effect of circ0120816 on ESCC tumor growth and metastasis, the xenograft mice model was constructed.

**Results:**

Experimental investigations revealed that circ0120816 was the highest upregulated circRNA in ESCC tissues and that this non-coding RNA acted as a miR-1305 sponge in enhancing cell viability, cell proliferation, and cell adhesion as well as repressing cell apoptosis in ESCC cell lines. Moreover, miR-1305 was observed to exert a tumor-suppressive effect in ESCC cells by directly targeting and repressing TXNRD1. It was also noticed that TXNRD1 could regulate cyclin, cell adhesion molecule, and apoptosis-related proteins. Furthermore, silencing circ0120816 was found to repress ESCC tumor growth and metastasis in vivo.

**Conclusions:**

This research confirmed that circ0120816 played an active role in promoting ESCC development by targeting miR-1305 and upregulating oncogene TXNRD1.

## Background

Esophageal cancer (EC) refers to the malignant growth found in the hollow tube connecting the throat to the stomach. In 2018, 572,034 new cases and 508,585 deaths were associated with EC worldwide [1]. Among them, 90% were identified as esophageal squamous cell carcinoma (ESCC) [2]. The incidence of ESCC was discovered to be extremely high in China: It accounted for about 50% of EC recorded globally [3, 4]. In the last 10 years, advanced strategies have been developed to treat multiple cancers, such as surgical operations, radiotherapy and chemotherapy [5–11]. However, compared with patients with other types of cancers, patients with ESCC had a high mortality rate despite the recent improvements in ESCC treatments [12, 13]. Also troubling is that the response of patients with ESCC to surgical operations or chemoradiotherapy had been poor, in part because of inadequate early diagnosis as well as the diffuse and invasive nature of this cancer [14, 15]. This unpalatable trend indicates an urgent need for effective molecular target approaches for ESCC therapy [12, 16].

Circular RNAs (circRNAs) can be referred to as non-coding RNAs with a circular structure [17–20]. These RNAs are found in the cytoplasm and are formed when two back-splicing sites (3′ and 5′ ends) are joined to form a covalently linked loop [21–23]. Recent research has confirmed that circRNA can regulate the development of several cancers, such as oral carcinoma, ovarian carcinoma, pancreatic carcinoma, hepatocellular carcinoma and bladder carcinoma [24–30]. It was also discovered in the literature that circRNA acted as a sponge for microRNAs (miRNAs), thereby affecting the development of carcinoma [31–33]. For instance, circ0000515 acted as a ceRNA of miR-326, thus increasing the expression of ELK1 and promoting ovarian carcinoma [34]. Only a few studies, nonetheless, have explored the impact of how circRNA can participate in the occurrence and development of ESCC by regulating miRNA. Stated another way, many studies cannot demonstrate how circRNA can regulate genes related to encoding proteins, let alone specify the regulatory function of circ0120816 in ESCC via its interaction with miRNA.

In the last 10 years, miRNA have been demonstrated to participate actively in human tumorigenesis [35–38]. More specifically, studies have found miR-203 to possess tumor-suppressive function by regulating downstream protein-coding genes in gastric cancer, lung cancer, breast cancer and ESCC [39–42]. Several research reports have documented that miR-34 can play crucial roles in multiple cancers by targeting biological systems that regulate cell proliferation and cell apoptosis [43–46]. Apart from that, miR-21 has also been observed to contribute to the progression of carcinomas. This microRNA targeted multiple essential components of p53, TGF-β and mitochondrial apoptosis tumor-suppressive pathways [47–49]. Besides, studies on miR-155 have revealed the crucial function of miR-155 in the tumorigenesis of such cancers as breast cancer, colon cancer and ESCC [50–52]. A newly identified miRNA, miR-1305 was reported to have lower expression in various cancers such as lung carcinoma, breast cancer and hepatocellular carcinoma [53–55]. Nevertheless, no studies have reported the involvement of miR-1305 in ESCC development or its ability to do so by increasing TXNRD1 expression. The interaction between circ0120816 and miR-1305 in ESCC development also remains unclear.

Thioredoxin reductase 1 (TXNRD1) encodes a protein that belongs to the pyridine nucleotide-disulfide oxidoreductase family. This protein is a member of the thioredoxin (Trx) system, and it plays a key role in redox homeostasis [56, 57]. TXNRD1 was widely reported to participate in the positive regulation of hepatocellular carcinoma [58–60]. In addition, TXNRD1 was observed to contribute to the development of lung cancer [61] and breast cancer [62, 63]. However, researchers are yet to provide detailed explanations regarding the relationship between TXNRD1 and ESCC.

This study aimed to explore the molecular mechanism underlying ESCC pathogenesis and investigate the function and interaction of circ0120816, miR-1305 and TXNRD1 in ESCC cell lines. This research is worthwhile in that the outcome might provide new insights into ESCC prognosis and treatments.

## Materials and methods

### Bioinformatics analysis

The GSE131969 downloaded from GEO DataSets was employed to screen the upregulated circRNAs, while GSE33810 and GSE20347 downloaded from GEO DataSets were used to identify the upregulated differentially expressed genes (DEGs). The upregualted circRNAs and the DEGs with adj. *P* value < 0.05 and log fold change (logFC) > 1.5 were selected in this study. STRING algorithm was utilized to analyze the key biological processes for DEGs. TargetScan and circInteractome analyses were later carried out to predict the miRNAs that could bind to TXNRD1 and circ0120816, respectively.

### Patients collection

A total of 36 patients from Wuhan Asia Heart Hospital Affiliated to Wuhan University of Science and Technology participated in this study. ESCC tissues and corresponding adjacent healthy tissues from these 36 ESCC patients were collected and used to explore the research objectives. Before data collection, informed consent was obtained from all the participants. The collection and usage of tissue samples were performed according to the ethical standards set out in the Helsinki Declaration and approved by the Ethical Committee of Wuhan Asia Heart Hospital Affiliated to Wuhan University of Science and Technology. The clinical characteristics of the 36 patients are shown in Table 1.

**Table 1:** Correlation between circ0120816 expression and clinical features of ESCC patients

Characteristics	Total = 36	Expression of circ0120816		*P* value^a^
		High (n = 20)	Low (n = 16)	
Age(years)				0.765
≤ 60	17 (47.2%)	9 (45.0%)	8 (50.0%)	
> 60	19 (52.8%)	11 (55.0%)	8 (50.0%)	
Gender				0.940
Female	16 (44.4%)	9 (45.0%)	7 (43.8%)	
Male	20 (55.6%)	11 (55.0%)	9 (56.2%)	
Tumor location in esophagus				0.491
Upper	8 (22.2%)	3 (15.0%)	5 (31.3%)	
Middle	17 (47.2%)	10 (50.0%)	7 (43.7%)	
Lower	11 (30.6%)	7 (35.0%)	4 (25.0%)	
Pathological T stage				0.020^b^
T1	10 (27.8%)	2 (15.0%)	8 (43.8%)	
T2	16 (44.4%)	10 (45.0%)	6 (43.8%)	
T3	10 (27.8%)	8 (40.0%)	2 (12.5%)	
Lymph node metastasis				
No	21 (58.3%)	7 (35.0%)	14 (87.5%)	0.001^b^
Yes	15 (41.7%)	13 (65.0%)	2 (12.5%)	
Differentiation				0.285
Well	7 (19.4%)	3 (25%)	4 (7.4%)	
Moderately	20 (55.6%)	10 (55%)	10 (51.9%)	
Poorly	9 (25.0%)	7 (20%)	2 (40.7%)	

^a^*P* value comparison between higher and lower circ0120816 expression (cut by the mean level) in ESCC tumor tissues; ^b^*P* < 0.05

### RNA extraction, reverse transcription and real-time quantification PCR

The dissociation of the RNAs was performed with the TaKaRa MiniBEST Universal RNA Extraction Kit (TaKaRa, Japan), and the RNAs were quantified using NanoDrop 2000 (Thermo Fisher Scientific, USA). After that, 1 μg RNA was reverse-transcribed with the PrimeScript II 1st Strand cDNA Synthesis Kit (TaKaRa, Japan). The 7500 Fast Dx Real-Time PCR Instrument (ABI, USA) was eventually used to measure the expression of circRNA, miRNA and mRNA in ESCC tissues and cell lines. The tumor tissues of the xenograft mice model were examined with the SYBR Green PCR Kit (Takara, Japan). GAPDH was used as the reference gene for circRNA and mRNA, whereas U6 was utilized as the reference gene for miRNA. Designed and synthesized from Tiangen Biochemical Technology (Beijing, China), all the primers used in this study are shown in Table 2.

**Table 2:** The primer sequences for RT-qPCR

Name	Primer sequences (5′-3′)
Circ_0120816	
Forward	AGCCAGAGTCTGTCGTGAAC
Reverse	TCCCACACCAGCAGAATCAT
Circ_0139153	
Forward	TGGGATTTTGCCTTTTGGTA
Reverse	CAGTGAATGGAATGCACCAG
Circ_0096710	
Forward	AGCAACATTTGGGGTTCATC
Reverse	GTTCGGCACATGGGTAAAAG
Circ_0095414	
Forward	TTATGATCACCCAGGGAGGA
Reverse	CTCCATTTCCACCTCCAGAA
Circ_0085539	
Forward	TAGATCCTGCCCTGTTTGCT
Reverse	CCACAGTGACAGCAGGACTC
miR-1305	
Forward	ACAGGCCGGGACAAGTGCAATA
Reverse	GCTGTCAACGATACGCTACGTAACG
U6	
Forward	CTCGCTTCGGCAGCACA
Reverse	AACGCTTCACGAATTTGCGT
TXNRD1	
Forward	AAATTCTTAGGACGGTCGGG
Reverse	AGTCTGCCCTCCTGATAAGC
GAPDH	
Forward	GTCAAGGCTGAGAACGGGAA
Reverse	AAATGAGCCCCAGCCTTCTC

### Cell culture

The human ESCC cell lines (KYSE30, KYSE180, KYSE450 and KYSE510) and the normal esophageal epithelial cell line Het-1A were purchased from Deutsche Sammlung von Mikroorganismen und Zellkulturen (Germany) and the STR profiling have been done for all the cell lines. All the ESCC cell lines were cultured in an RPMI 1640 medium, which had been supplemented with 10% FBS and 1% streptomycin/penicillin in a humidified incubator containing 5% CO_2_ at 37 °C. The mycoplasmas in the culture cells were tested once in 3 months.

### Cell transfection

The circ0120816 siRNA (Si-circ0120816), circ0120816 shRNA (Sh-circ0120816), circ0120816 overexpression plasmid (OE- circ0120816), miR-1305 mimic, miR-1305 inhibitor, TXNRD1 siRNA (si-TXNRD1) and their negative control (NC) were designed by GeneCopoeia (Guangzhou, China). Lipofectamine 2000 Transfection Reagent (Thermo Fisher Scientific, USA) was used to conduct cell transfection. KYSE450 or KYSE510 cells at the exponential growth stage were digested with trypsin and seeded in a 6-well plate at a density of 2 × 105 cells/well. The cell transfection process was then carried out when the density of KYSE450 or KYSE510 cells reached 70–80%. As described in the protocol of Lipofectamine 2000 Transfection Reagent, 50 nM Si-circ0120816, miR-1305 mimic, miR-1305 inhibitor, si-TXNRD1 or NC was transfected into KYSE450 and KYSE510 cells. After 48-h transfection, qRT-PCR was used to analyze the transfection efficiency.

### RNase R degradation assay

After KYSE450 or KYSE510 cells were extracted, the total RNAs were inactivated with an RNase inhibitor (Beijing Tiangen Biochemical Technology, China) at 37 °C for 15 min. An equal RNA was then used to perform reverse transcription into cDNA, and qRT-PCR was utilized to analyze the expression of circ0120816. This procedure was done to assess the stability of circ0120816 and its linear isoform. Three independent repeats were conducted for each set of the RNase R treatment experiment.

### CircRNA subcellular localization assay

The cytoplasmic and nuclear components were first extracted using the Nuclear Extraction Kit (Millipore, USA). The manufacturer’s guideline was followed religiously: 1 × 10^7^ cells were collected and supplemented with warmed trypsin cell detachment buffer, and the samples were then incubated for 2 min. Ice-cold 1 × Cytoplasmic Lysis Buffer, which contained 0.5 mM DTT, was added to the sample before incubating it on ice for 15 min. The cytoplasmic fraction was collected after 3-min centrifugation at maximum speed. As for the nuclear extraction, the nuclear pellet was resuspended in the cell pellet volume in an ice-cold Nuclear Extraction Buffer. A rotator was subsequently used to agitate the nuclear suspension gently at 4 °C for 60 min. The nuclear suspension centrifuged at 16,000 × g for 5 min at 4 °C, as well as the supernatant, was collected for the nuclear extract. Finally, the cytoplasmic fraction and nuclear extract were subjected to RNA extraction and RT-qPCR with or without RNase R treatment. This extraction was done to determine the subcellular localization of the circular and linear form of circ0120816. Three independent repeats were conducted for each set of the circRNA subcellular localization assay.

### Luciferase reporter assay

The wild and mutant circ0120816 sequences or wild and mutant TXNRD1 3′UTR sequences synthesized from GeneCopoeia (Guangzhou, China) were subcloned into the pmiR-GLO reporter vector (circ-WT, TXNRD1-WT or circ-Mut, TXNRD1-Mut). After that, 5 × 10^3^ per well of KYSE450 or KYSE510 cells were seeded in a 96-well plate and incubated for 24 h. Next, the samples were co-transfected with 100 ng of circ-WT or circ-Mut (TXNRD1-WT or TXNRD1-Mut) and 50 nM of miR-1305 mimic or mimic NC. After 48-h co-transfection, the cells were collected and lysed. The Dual-GLO® Luciferase Assay System Kit (Promega, USA) and Fluorescence/Multi-Detection Microplate Reader (BioTek, USA) were eventually used to detect luciferase activities. Three independent repeats were conducted for each set of the luciferase reporter assay.

### RNA immunoprecipitation assay (RIP assay)

RNA immunoprecipitation assay was performed to identify the relationship between circ0120816 and miR-1305. The Magna RIP RNA-Binding Protein Immunoprecipitation Kit (Millipore, USA), which was used in accordance with the user guide, was used to identify the relationship between circ0120816 and miR-1305. 1 × 10^6^ KYSE450 or KYSE510 cells were lysed in the RIP lysis buffer. The cell lysates were then incubated with magnetic beads conjugated with human anti-Argonaute2 (Ago2) antibody or negative control IgG at 4 °C for 12 h. After that, the sample was treated with Proteinase K for 30 min at 37 °C. Finally, the magnetic beads were washed twice with the RIP buffer, and the immunoprecipitated RNA was isolated with the TaKaRa MiniBEST Universal RNA Extraction Kit (TaKaRa, Japan). After RT-qPCR analysis, circ0120816 expression in the immunoprecipitated RNA was detected. Three independent repeats were conducted for each set of the RIP assay.

### CCK-8 assay

Cell Counting Kit-8 (Vazyme, China) was used to detect cell viability. KYSE450 or KYSE510 cells were seeded in a 96-well plate at a density of 1 × 10^4^ cells/well. At different periods (0, 24, 48 and 72 h), 10 μL of CCK-8 solution was added to each well of the plate using a repeating pipettor. The plate was incubated in the dark for 1 h. The absorbance at 450 nm was measured using a microplate reader (BioTek, USA). Three independent repeats were conducted for each set of the CCK-8 assay.

### BrdU assay

The proliferation of ESCC cells was analyzed using the BrdU Cell Proliferation Assay Kit (Cell Signaling Technology, USA). Cell culture was carried out on 96-well plates with a cell density of 1 × 10^4^ cells/well in 100 μL culture media. This procedure was performed according to the standard protocol recommended by the manufacturer. The cell wells were later supplemented with 10 μL 10 × BrdU labeling solution and then incubated at 37 °C for 24 h. Next, the culture media were replaced with the Fixing/Denaturing Solution (100 μL) and then incubated at room temperature for 30 min. Afterward, the plates were washed three times with 1 × Wash Buffer and supplemented with 100 μL prepared 1 × Detection Antibody solution for 1 h incubation at room temperature. Subsequently, the plates were washed again, and 100 μL 1 × HRP-conjugated secondary antibody solution and 100 μL TMB substrate were in turn added and incubated for 30 min at room temperature. Finally, 100 μL STOP Solution was added to the cell wells, and the absorbance at 450 nm was measured in a microplate reader (BioTek, USA). Three independent repeats were conducted for each set of the BrdU assay.

### Cell adhesion assay

Type I collagen (BD Bioscience, CA) was first coated on the 96-well plates. This was done according to the manufacturer's guideline. Then, 50 μL 10 μg/mL type I collagen was added to each well of the plates. After that, the 96-well plates were incubated at 37 °C for 1 h. Next, the transfected KYSE450 or KYSE510 cells were digested with trypsin, suspended in a serum-free culture medium, and seeded in a 96-well plate precoated with collagen I solution at 5 × 10^4^ cells per well. After 1-h incubation at 37 °C, PBS was added to remove unwanted cells. Subsequently, 100 µL 10% ethanol was added to each well and incubated for 5 min at 25 °C. The absorbance was finally determined using a microplate reader (BioTek, USA) at 570 nm. Three independent repeats were conducted for each set of the cell adhesion assay.

### Cell cycle assay

The Propidium Iodide Flow Cytometry Kit (Abcam, USA) was used to analyze the cell cycle of KYSE450 and KYSE510 cells. According to the product protocol, the transfected KYSE450 and KYSE510 cells were first digested with trypsin to obtain single-cell suspension. Then, the cells were fixed with 66% ethanol at 4 °C for 2 h. After that, the samples were washed twice with PBS. After 5-min centrifugation at 500 × *g*, the cell pellets were obtained and resuspended in prepared 1 × Propidium Iodide + RNase Staining Solution. The samples were then incubated at 37 °C in the dark for 20 min. Finally, the stained cells were loaded to a flow cytometer for flow cytometry analysis. The DNA content represents the cell cycle phase localized by the cells. Three independent repeats were conducted for each set of the cell cycle assay.

### Western blot

The total protein in transfected KYSE450 and KYSE510 cells was extracted and quantified with the RIPA buffer (Solarbio, China), which was supplemented with 1% proteinase inhibitor cocktail. The protein concentration was obtained with the Pierce BCA protein assay kit (Thermo Fisher Scientific, USA). Next, the quantified protein was loaded into 10% or 12% SDS-PAGE for electrophoresis separation. The separated protein bands were then electronically transferred to the membranes (Sigma-Aldrich, USA). Following that, the membranes were put under ice for 2 h with 5% BSA at room temperature. This step was followed by the incubation of the membranes overnight with primary antibodies at 4 °C and 1.5 h incubation with secondary detection antibody (Cat# ab205718, Abcam, USA) at room temperature. Finally, the protein immunoblots were visualized using the Enhanced Chemiluminescent (ECL) Reagent Kit (Thermo Fisher Scientific. USA). The density of the blots was obtained using ImageJ software. All the primary antibodies were purchased from Abcam (USA), including anti-CyclinB1 (Cat# ab32053), anti-ICAM1 (Cat# ab53013), anti-VCAM1 (Cat# ab134047), anti-Cleaved PARP (Cat# ab32561), anti-Bax (Cat# ab32503), anti-Cleaved Caspase-3 (Cat# ab2302), anti-TXNRD1 (Cat# ab124954) and anti-β-actin (Cat# ab8227).

### Caspase 3 activity assay

The Caspase-3 Activity Assay Kit (Cell Signaling, USA) was employed to detect the activities of caspase-3 in KYSE450 and KYSE510 cells. In brief, the cells were seeded in a 96-well plate at a density of 5 × 10^3^/well. After the transfection process, the cells were washed twice with ice-cold PBS. Subsequently, 30 μL cell lysis buffer was added to each well, and the culture plate was placed on the ice for 5 min. The lysates were then treated with ultrasound on ice and separated using microcentrifugation at 4 °C for 10 min. Next, the lysate solution (25 µL) was obtained and mixed with 200 µL substrate solution B on a black culture plate suitable for fluorescence detection. This step was followed by 60 min incubation in the dark. Finally, the absorbance was measured using a microplate at 450 nm. Three independent repeats were conducted for each set of the caspase-3 activity assay.

### Tumor xenograft mice model construction

SPF-grade BALB/c nude mice at 6 weeks were purchased from SPF Biotechnology Co., Ltd. (Beijing, China) and housed in a 12/12 h light/dark cycle in a pathogen-free animal facility at Wuhan University of Science and Technology. After 1 week housing, the nude mice were randomly divided into two groups (3 mice in each group). The mice were then subcutaneously injected with 1 × 10^6^ KYSE450 cells transfected with circ0120816 shRNA. Other mice that were not injected were used as the negative control. At a xenograft period of 1 and 4 weeks, the xenograft mice were intraperitoneally injected with 75 mg/kg luciferin substrate D-luciferin potassium salt (Shanghai, China). The IVIS 200 bioluminescence imaging system (Caliper Life Sciences, Hopkinton, MA) was also used to quantify the fluorescence and bioluminescence marks in the mice. The mice were then euthanized, and the tumor tissues were obtained for further RT-qPCR and histological staining analysis. For the in vivo lung metastasis examination, 1 × 10^6^ KYSE450 cells transfected with circ0120816 shRNA or negative control were tail intravenously injected into the nude mice (3 mice in each group), respectively. After housing them for 4 weeks, the mice were euthanized, and the lungs tissues of mice were collected for histological staining. All the animal experiments were approved by the Animal Welfare and Research Ethics Committee of Wuhan University of Science and Technology.

### Hematoxylin and eosin (H&E) staining

Before H&E staining, the fresh collected mice tissues with 4% polyformaldehyde were fixed for 18 h. The fixed tissues were subsequently subjected to gradient alcohol dehydration, xylene hyalinization and paraffin embedding. Next, 5 μm-thick tissue sections were obtained by cutting the paraffin-embedded tissues in a rotary microtome. After that, the tissue sections underwent 15-min de-wax with xylene, 30-min hydration with gradient alcohol, and 2-min staining with hematoxylin staining. This step was followed by the treatment of the samples with 1% hydrochloric acid alcohol solution. Finally, the tissue sections were stained with eosin for 10 s, dehydrated with gradient alcohol and mounted with neutral balsam. The images of stained tissue sections were captured with the microscope camera (Leica, Germany).

### RNA pull-down

MiR-1305 mimic-biotin (Bio-miR-1305) and its negative control (Bio-NC) were synthesized from RiboBio (Guangzhou, China). The Pierce Magnetic RNA–Protein Pull-Down Kit (Thermo Scientific, USA) was used to perform RNA pull-down. 5 × 10^5^ KYSE450 and KYSE510 cells were first seeded in the 6-well plates and then kept in an incubator overnight. Then the Bio-miR-1305 or Bio-NC were transfected for 48 h using Lipofectamine 2000 Transfection Reagent. Next, the cell lysis buffer was blended with probe-bead complex and added to the transfected cells for 3 h incubation at 4 °C. After that, the reaction tubes were placed on the magnetic stand for the collection of the beads. Protein K and DNase A were later used to remove the protein and DNA, respectively. Finally, the RNA in RNA-bead complex was eluted using the RNeasy Mini Kit (QIAGEN) and was reversed transcript to cDNA. Finally, the TXNRD1 mRNA expression level was measured using qRT-PCR. Three independent repeats were performed for each set of the RNA pull-down assay.

### Statistical analyses

Data analysis was performed with SPSS 23.0 (SPSS, USA) and GraphPad Prism 8.0 (GraphPad Software, USA). All data used for analysis were obtained from three independently repeated experiments. The data were represented in the form of mean ± standard deviation (SD). Student’s *t*-test was used to analyze the statistical differences between two groups, while ANOVA was employed to examine the statistical differences among multiple groups. The chi-squared test was utilized to obtain the correlation between the expression of circ0120816 and to determine the clinical features of ESCC patients. P-values less than 0.05 were regarded as statistically significant.

## Results

### Identification of circ0120816/miR-1305/TXNRD1 axis as the key regulator in ESCC

**Fig. 1:**
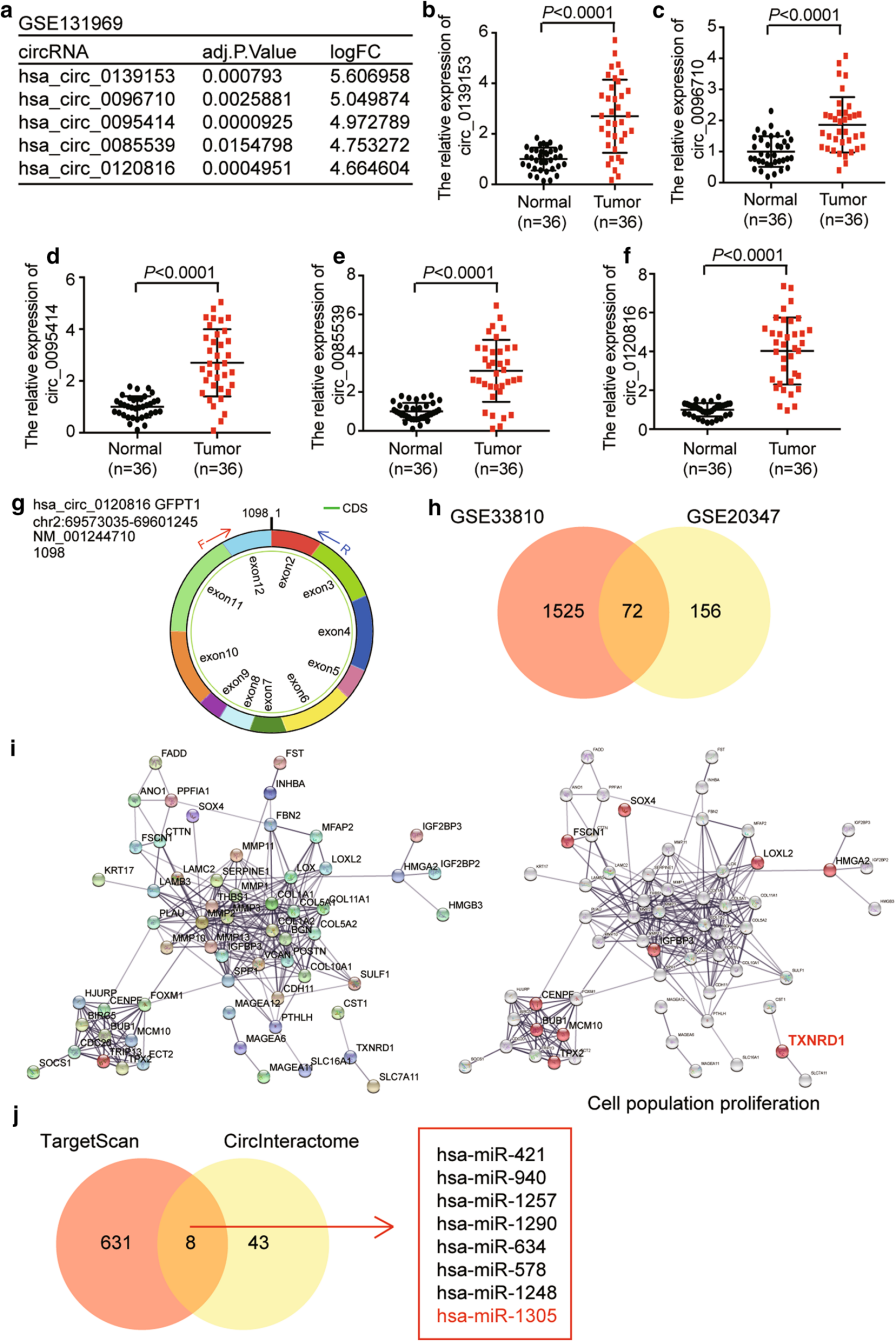
The identification of circ0120816/miR-1305/TXNRD1 axis in ESCC. **a** Microarray analysis revealed the top 5 upregulated circRNAs in circRNA microarray GSE131969. **b**–**f** RT-qPCR analysis showed the expression of the top 5 upregulated circRNAs in ESCC clinical samples. **g** The structure of circ0120816. **h** A total of 72 upregulated DEGs were overlapped in datasets of GSE33810 and GSE20347 using Venny 2.1.0 analysis. **i** TXNRD1 was the key gene involved in cell population proliferation. **j** Eight miRNAs were identified to be overlapped using TargetScan and circInteractome analyses. TargetScan was utilized to predict miRNAs binding to TXNRD1. CircInteractome was employed to predict miRNAs binding to circ0120816

GSE131969 was downloaded from the GEO DateSets and was used to confirm the key circRNA participating in ESCC progression. The top 5 upregulated circRNAs are shown in Fig. 1a. After detecting the expression of the top 5 upregulated circRNAs in the 36 paired clinical ESCC tissue samples, circ0120816 with the highest expression in tumor samples was observed compared with other circRNAs (Fig. 1b–f). Therefore, the circRNA identified to be explored in this study was circ0120816. The structure of circ0120816 is shown in Fig. 1g. GSE33810 and GSE20347, which were downloaded from the GEO DateSets, were used to screen the key genes associated with ESCC. By employing Venny.2.1.0 analysis, 72 upregulated DEGs were found to be overlapped in GSE33810 and GSE20347 (Fig. 1h). After uploading the 72 common genes to STRING, the potential interactome of these genes was obtained. TXNRD1, one of the proliferation-related genes, was hypothesized to play a regulatory role in ESCC progression (Fig. 1i). To confirm the key miRNA linking circ0120816 and TXNRD1 in ESCC, TargetScan and circInteractome were used to predict the miRNAs bound to TXNRD1 and circ0120816, respectively. Eight miRNAs were eventually predicted using TargetScan and circInteractome (Fig. 1j). After researching the literature, miR-1305 was identified as the target miRNA because of its tumor-suppressor effect on multiple cancers [53, 55, 64, 65].

### Upregulation of circ0120816 in ESCC cells

**Fig. 2:**
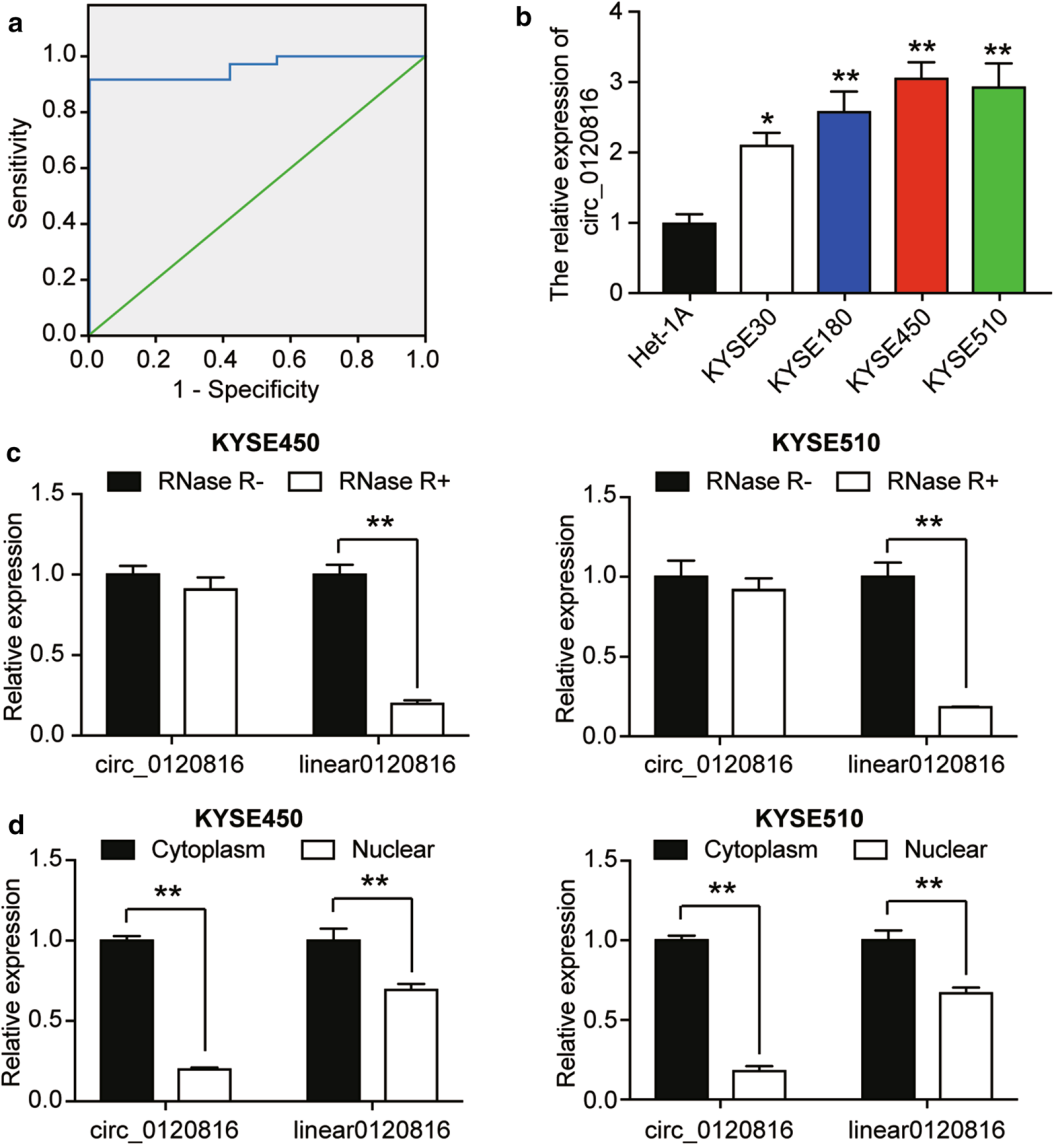
Circ0120816 was upregulated in ESCC cells. **a** The receiver operating characteristic (ROC) curve showed the diagnostic value of circ0120816 for ESCC. The ROC curve was determined based on the circ0120816 expression in ESCC tissues and adjacent normal tissues. **b** RT-qPCR analysis revealed the expression of circ0120816 in ESCC cell lines (KYSE30, KYSE180, KYSE450, KYSE510) and normal cells (Het-1A). *P < 0.05, **P < 0.001, compared with Het-1A cells. **c** RNase R degradation assay confirmed the stability of circ0120816. **P < 0.001, the comparison between RNase R- and RNase R + . **d** The distribution of circ0120816 and linear 0120816 in ESCC cells was determined by circRNA subcellular localization assay. **P < 0.001, the comparison between the cytoplasm and the nucleus. Each cellular experiment was independently repeated three times, and the data collected were displayed in the format of mean ± standard deviation (SD)

We used the receiver operating characteristic (ROC) curve to determine the potential of circ0120816 in ESCC diagnosis. Our result indicated that the expression of circ0120816 could effectively distinguish ESCC from normal tissues, thus suggesting that circ0120816 might improve the diagnosis, sensitivity and specificity of ESCC combined with other traditional tumor markers (Fig. 2a). Moreover, circ0120816 was discovered to be significantly associated with the occurrence of ESCC clinical characteristics, including pathological T stage and lymph node metastasis (Table 1). To observe the difference of circ0120816 expression between ESCC cells (i.e., KYSE30, KYSE180, KYSE450, and KYSE510) and normal human esophageal epithelial cell, we designed and synthesized the divergent primers for qRT-PCR. We found that the expression of circ0120816 in ESCC cells was significantly higher than that in the esophageal epithelial cell line (Het-1A) (Fig. 2b). What’s more, circ0120816 expression in KYSE450 and KYSE510 was higher in KYSE450 and KYSE510 than in other ESCC cells. For this reason, we used KYSE450 and KYSE510 cells for further experimental investigations. RNase R degradation assay was later used to determine the stability of circ0120816 in ESCC cells. The results showed that circ0120816 was resistant to RNase R treatments, while linear 0120816 was dramatically degraded by around 70% (Fig. 2c). In order to ascertain the specific role of circ0120816 in ESCC cells, the sub-localization of circ0120816 in KYSE450 and KYSE510 cells was determined with the circRNA subcellular localization assay. The RT-qPCR results indicated that both circular and linear type of circ0120816 were found mainly in the cytoplasm of ESCC cells (Fig. 2d). More specifically, circ0120816 expression in the cytoplasm was five-fold as high as the 0120816 expression in nuclear (Fig. 2d).

### MiR-1305 was the downstream gene of circ0120816

**Fig. 3:**
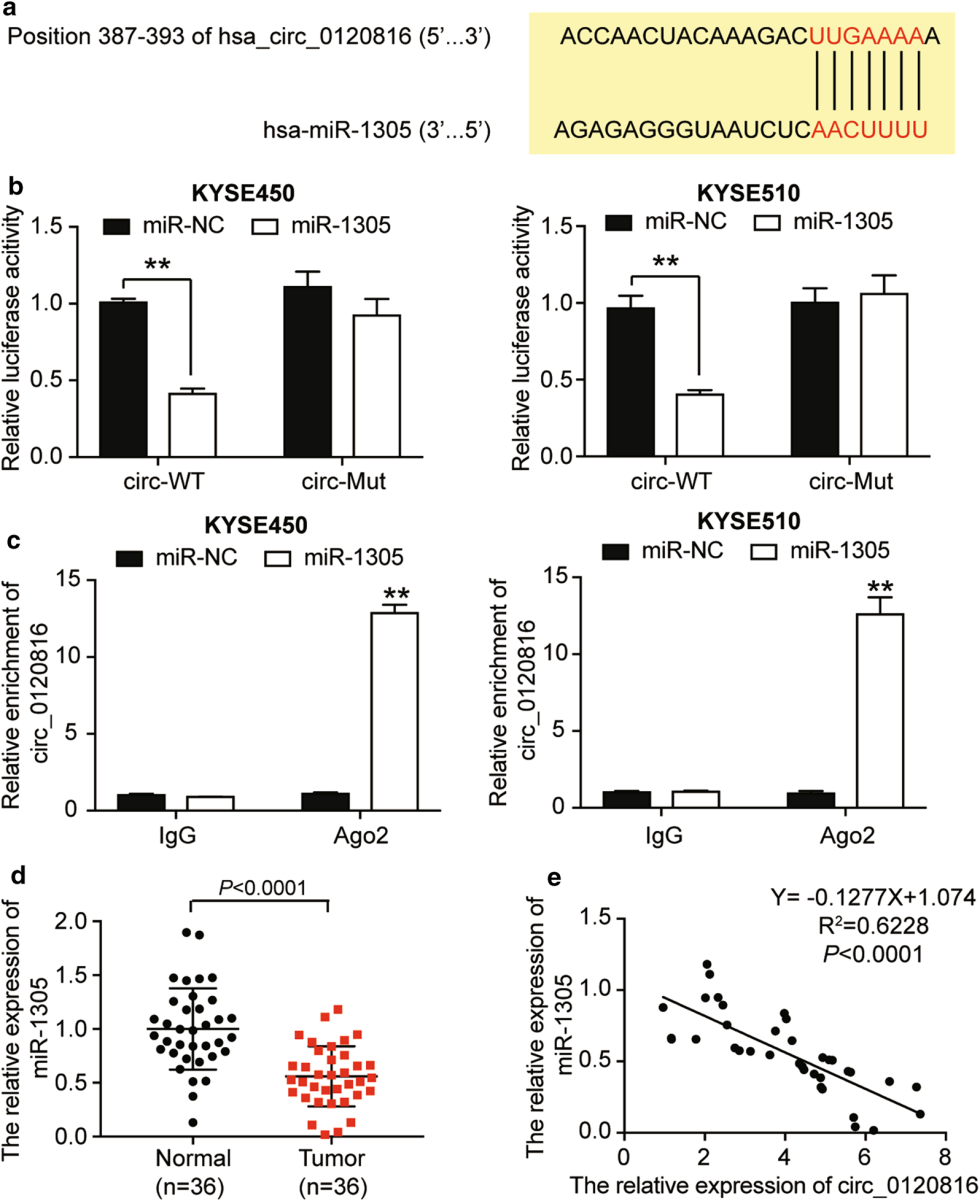
Circ0120816 acted as a sponge for miR-1305. **a** The binding sequence between circ0120816 and miR-1305 was predicted by TargetScan 7.0. **b** Luciferase reporter assay was used to confirm the relationship between circ0120816 and miR-1305. **P < 0.001, compared with miR-NC. miR-NC: miR-1305 negative control, circ-WT: circ0120816 wild type, circ-Mut: circ0120816 mutant type. **c** RIP analysis showed that circ0120816 was abundantly pulled down by antiAgo2 antibodies when transfected with miR-1305 mimics in ESCC cells compared with the miR-1305 NC and IgG group. **P < 0.001, compared with miR-NC. miR-NC: miR-1305 negative control, IgG: negative control, Ago2: anti-Argonaute2 antibody. **d** RT-qPCR analysis showed that the expression of miR-1305 was decreased in ESCC tissues. Normal: adjacent healthy tissues, Tumor: ESCC tissues. **e** Pearson’s correlation analysis showed that miR-1305 was negatively associated with circ0120816. Each cellular experiment was independently repeated three times, and the data collected were illustrated in the format of mean ± standard deviation (SD)

Research has shown that circRNA exerts its function by sponging miRNA. To better understand the molecular mechanism of circ0120816 in ESCC progression, we explain its interaction with miRNA. CircInteractome was used to predict the binding site of circ0120816 with miR-1305 with the sequences showed in Fig. 3a. Next, we used a luciferase reporter assay to observe the relationship between circ0120816 and miR-1305. Results indicated that the luciferase activity was significantly decreased by about 60% in circ-WT and that miR-1305 co-transfected KYSE450 and KYSE510 cells compared to miR-NC groups even though no difference was found between circ-mutant co-transfection groups (Fig. 3b). Furthermore, RIP assay results displayed that circ0120816 significantly enriched cells transfected with miR-1305 mimic, which could be repressed by anti-Ago2 antibody (Fig. 3c). After performing RT-qPCR analysis, we observed that the expression of miR-1305 in tumor tissues with ESCC was quite low (Fig. 3d). Moreover, Pearson’s correlation analysis indicated that miR-1305 had a negative correlation with circ0120816 in ESCC tissues (Fig. 3e). Overall, findings confirmed that circ0120816 acted as a sponge for miR-1305 in ESCC upregulation, meaning it might play a critical role in ESCC development.

### Circ0120816 facilitated ESCC progression by sponging miR-1305

**Fig. 4:**
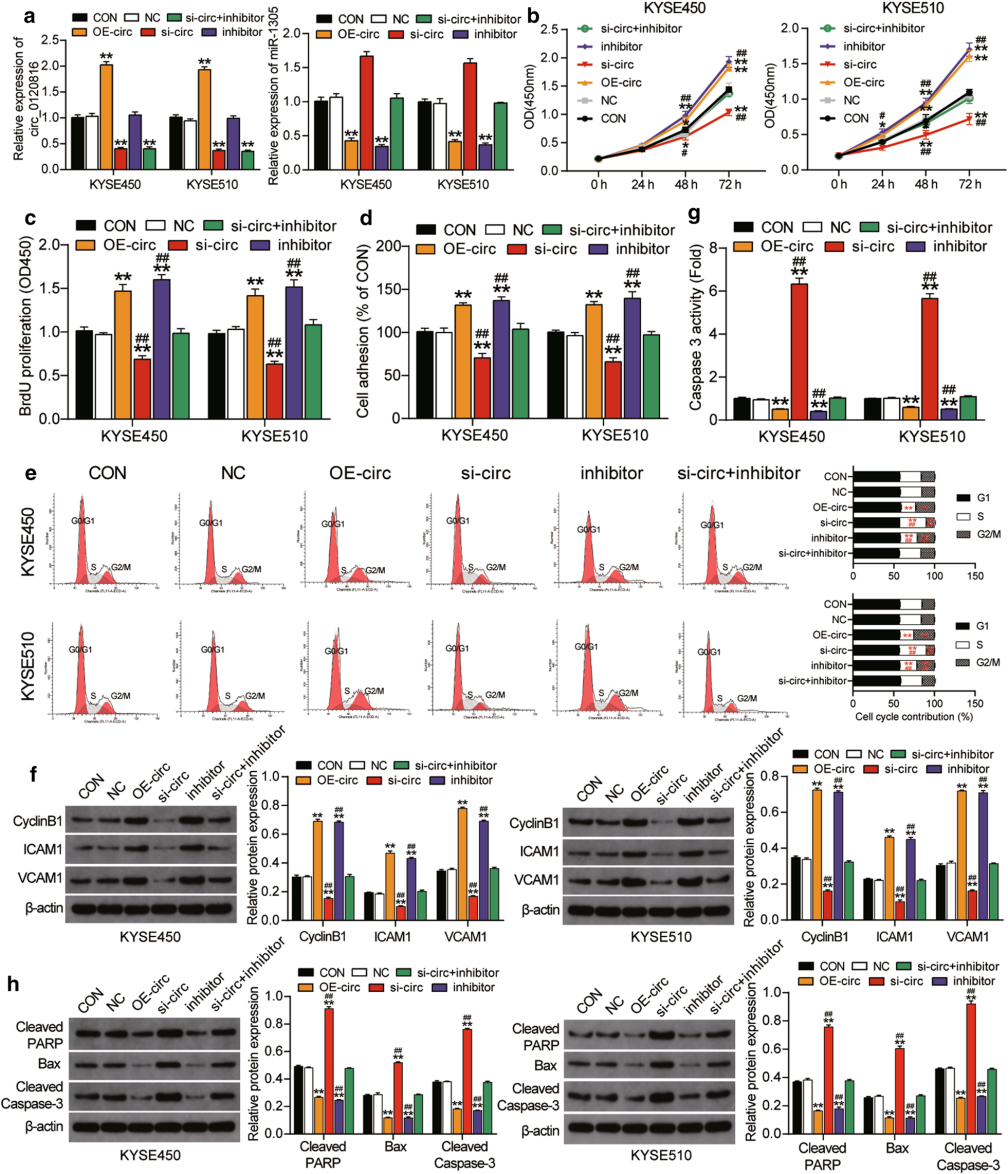
Circ0120816 facilitated ESCC progression by sponging miR-1305. **a** RT-qPCR analysis of the expression of circ0120816 and miR-1305 in ESCC cell lines after transfecting with circ0120816 overexpression plasmid, circ0120816 siRNA or miR-1305 inhibitor. **b** CCK-8 assay was used to determine the viability of ESCC cell lines after transfection. **c** BrdU assay was conducted to evaluate the proliferation of ESCC cell lines after transfection. **d** Cell adhesion assay was employed to determine the cell adhesion ability of ESCC cell lines after transfection. **e** Cell cycle assay was applied to evaluate the cell cycle in ESCC cell lines after transfecting with a flow cytometry. **f** The expression of cyclinB1, ICAM1 and VCAM1 was detected in ESCC cell lines after transfection by western blot assay. **g** Caspase 3 activity assay indicates the cell apoptosis of ESCC cell lines after transfection. **h** The expression of Cleaved PARP, Bax and Cleaved Caspase-3 was detected in ESCC cell lines after transfecting with western blot assay. *P < 0.05, **P < 0.001, compared with the blank control group. ^#^*P* < 0.05, ^##^*P* < 0.001, compared with the co-transfection group of si-circ0120816 plus miR-1305 inhibitor. CON: blank control, NC: negative control, OE-circ: circ0120816 overexpression plasmid, si-circ: circ0120816 siRNA, inhibitor: miR-1305 inhibitor, si-circ + inhibitor: circ0120816 siRNA plus miR-1305 inhibitor. Each experiment was independently repeated three times, and the data collected were displayed in the format of mean ± standard deviation (SD)

The impact of circ0120816 in ESCC progression was determined using several experiments. In each experiment, circ0120816 overexpression plasmid (OE-circ), circ0120816 siRNA (si-circ), miR-1305 inhibitor (inhibitor), circ0120816 siRNA and miR1305 inhibitor (si-circ + inhibitor) or the corresponding negative control (NC) were transfected into KYSE450 and KYSE510 cells with untreated cells as the blank control (CON). To determine the effect of transfection, we used RT-qPCR to measure the expression of circ0120816 and miR-1305. The result showed that the circ0120816 expression was increased in the OE-circ group by twofold and that it decreased in the si-circ group by 70% compared to the blank control group. However, no change in the miR-1305 inhibitor group was found. This experimental investigation indicated that circ0120816 expression was not regulated by the miR-1305 inhibitor (Fig. 4a). On the other hand, the miR-1305 was downregulated by about 60% in the OE-circ group, while it was upregulated by more than 1.5-fold in the si-circ group compared to the blank control group. Furthermore, miR-1305 was found to be reduced in the miR-1305 inhibitor group by about 70%. Besides, the miR-1305 inhibitor could effectively rescue the upregulation of miR-1305 caused by si-circ0120816 (Fig. 4a). CCK-8 assay results also showed that OE-circ0120816 miR-1305 inhibitor enhanced the cell viability of KYSE450 and KYSE510 cells, while si-circ0120816 inhibited it. However, the cell viability of ESCC cells co-transfected with si-circ0120816 and miR-1305 inhibitor did not show any significant differences compared with the control group (Fig. 4b). As for the BrdU assay results, we observed that si-circ0120816 could decrease cell proliferation by 40%, whereas OE-circ0120816 and miR-1305 inhibitor could increase cell proliferation by about 50% and 60%, respectively. Moreover, the ability of si-circ0120816 to perform cell proliferation could be compromised by co-transfecting the miR-1305 inhibitor in both KYSE450 and KYSE510 cells (Fig. 4c).

Our cell adhesion assay results showed that si-circ0120816 repressed cell adhesion by about 40%, while OE-circ0120816 and miR-1305 inhibitor facilitated cell adhesion by about 30% and 40%, respectively, compared to the blank control (Fig. 4d). Furthermore, the change caused by si-circ0120816 or miR-1305 inhibitor was reversed after the co-treatment of si-circ0120816 and miR-1305 inhibitor (Fig. 4d). The cell cycle assay results demonstrated that si-circ0120816 resulted in the S phase arrest in ESCC cells but that OE-circ0120816 and miR-1305 inhibitor significantly promoted ESCC cells to enter the G2/M phase. However, in KYSE450 and KYSE510 cells co-transfected with si-circ0120816 and miR-1305 inhibitor, the cell cycle showed no change compared to the blank control group (Fig. 4e). To further investigate how circ0120816 regulated ESCC cell cycle and adhesion, we detected the protein expression of cell cycle-related protein CyclinB1 and cell adhesion-related protein ICAM1 and VCAM1 in multiple groups. The western blot data demonstrated more than two-fold increase of these three proteins (CyclinB1, ICAM1 and VCAM1) in the OE-circ0120816 and miR-1305 inhibitor group, while it revealed about 50% decrease of these three proteins in the si-circ0120816 group compared to the control group. Moreover, the changed protein expression in the si-circ0120816 group could be completely reversed by co-transfecting the miR-1305 inhibitor (Fig. 4f). After we designed caspase 3 activity assay to further explore the effect of circ0120816 on cell apoptosis of ESCC cells, we found that si-circ0120816 facilitated cell apoptosis by about five-fold and OE–circ0120816 and miR-1305 inhibitor restrained cell apoptosis by about 50% and 60%. However, the consequence of si-circ0120816 or miR-1305 inhibitor could be compromised by si-circ0120816 and miR-1305 inhibitor co-treatment in both KYSE450 and KYSE510 cells (Fig. 4g). The protein level of pro-apoptotic proteins (i.e., Cleaved PARP, Bax and Cleaved Caspase-3) in multiple groups were later examined thoroughly to determine the mechanism used by circ0120816 to regulate ESCC cell apoptosis. The western blot results showed that OE-circ0120816 and miR-1305 inhibitor remarkably suppressed the expression of the three pro-apoptotic proteins, while si-circ0120815 significantly enhanced the expression of the three pro-apoptotic proteins. However, the enhanced protein expression caused by si-circ0120816 was completely compromised by co-transfecting the miR-1305 inhibitor (Fig. 4h). Overall, circ0120816 strengthened cell viability, proliferation and adhesion, but it repressed the apoptosis of ESCC cells in vitro by targeting miR-1305.

### Silencing circ0120816 inhibited the tumor growth and metastasis of ESCC in vivo

**Fig. 5:**
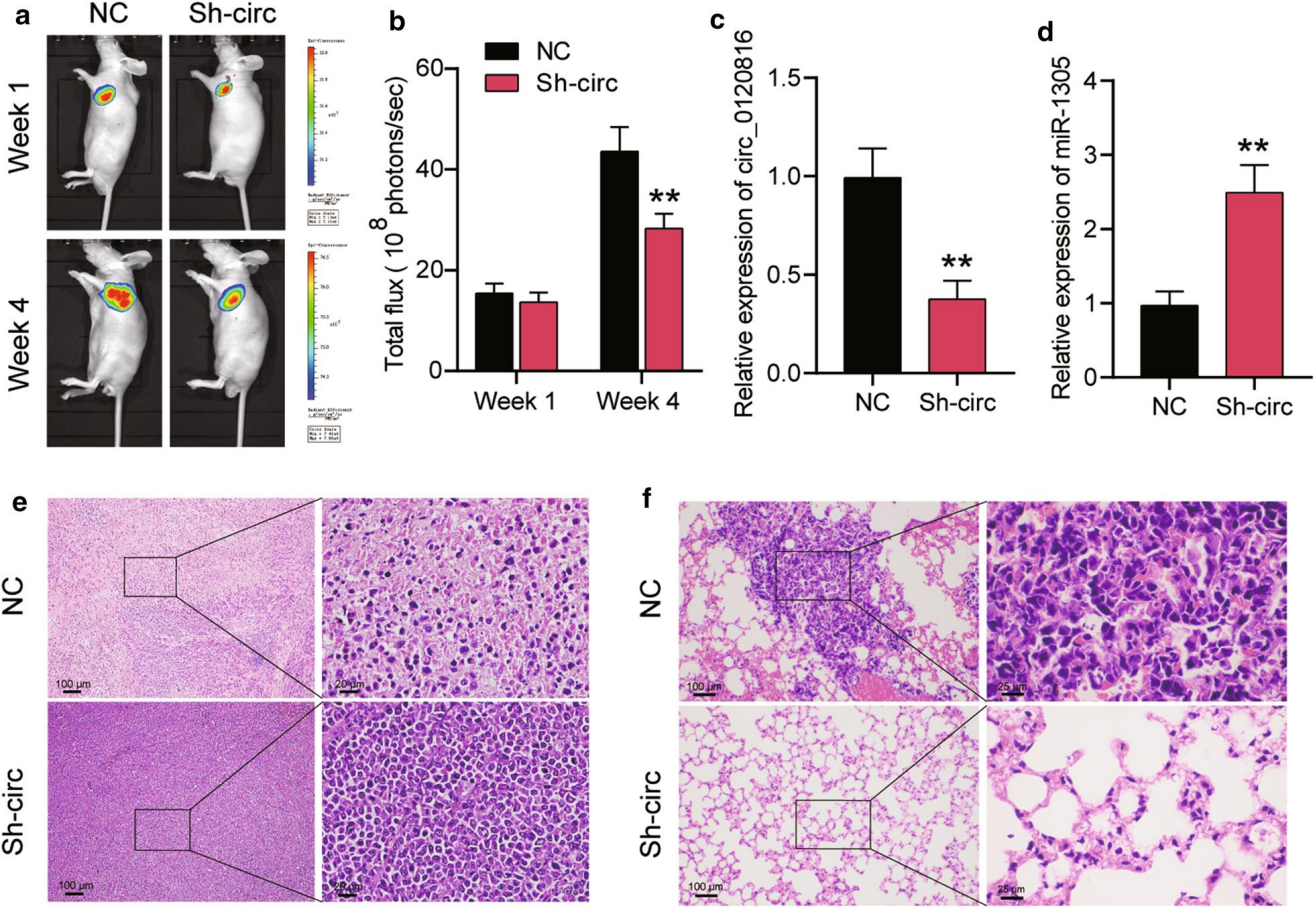
Silencing circ0120816 could inhibit the tumor growth and lung metastasis of ESCC in vivo. **a** Representative live imaging of xenograft mice with transplantation of KYSE450 cells lines pre-transfected with circ0120816 shRNA (Sh-circ) or negative control (NC) at week 1 and week 4. **b** Statistical analysis of the live imaging results. **c** RT-qPCR analysis of circ0120816 expression in the tumor tissues of xenograft mice. **d** RT-qPCR analysis of miR-1305 expression in the tumor tissues of xenograft mice. **e** Pathological H&E staining of tumor tissues collected from the Sh-circ and NC group mice. **f** Pathological H&E staining of lung tissues collected from the Sh-circ and NC group mice. **p < 0.001 compared with the NC group

To determine the effect of circ0120816 on ESCC tumor growth and metastasis in vivo, we constructed a xenograft tumor mice model by transplanting ESCC cell lines pre-transfected with circ0120816 shRNA (Sh-circ) or negative control (NC) into the BALB/c nude mice. The live cell imaging results indicated that the radiance flux of the mice transplanted with Sh-circ transfected KYSE450 cells showed a 35% reduction compared with NC mice after 4 week transplantation (Fig. 5a, b). It was also observed that the level of circ0120816 in the xenograft tumor tissues of Sh-circ group mice was downregulated by about 60% (Fig. 5c), while the level of miR-1305 was upregulated by 2.5-fold compared to the NC group mice (Fig. 5d). To further evaluate the impact of circ0120816 on ESCC progression, the pathological H&E staining was performed, and the results revealed a significantly reduced necrosis phenotype in the tumor tissues of the Sh-circ group mice compared to the NC group mice (Fig. 5e). Also, attenuated lung metastasis was found in the Sh-circ group mice (Fig. 5f). Taken together, these results indicated that circ0120816 was an oncogenic factor of ESCC and that silencing circ0120816 suppressed the tumor growth and metastasis of ESCC in vivo.

### TXNRD1: a target gene of miR-1305

**Fig. 6:**
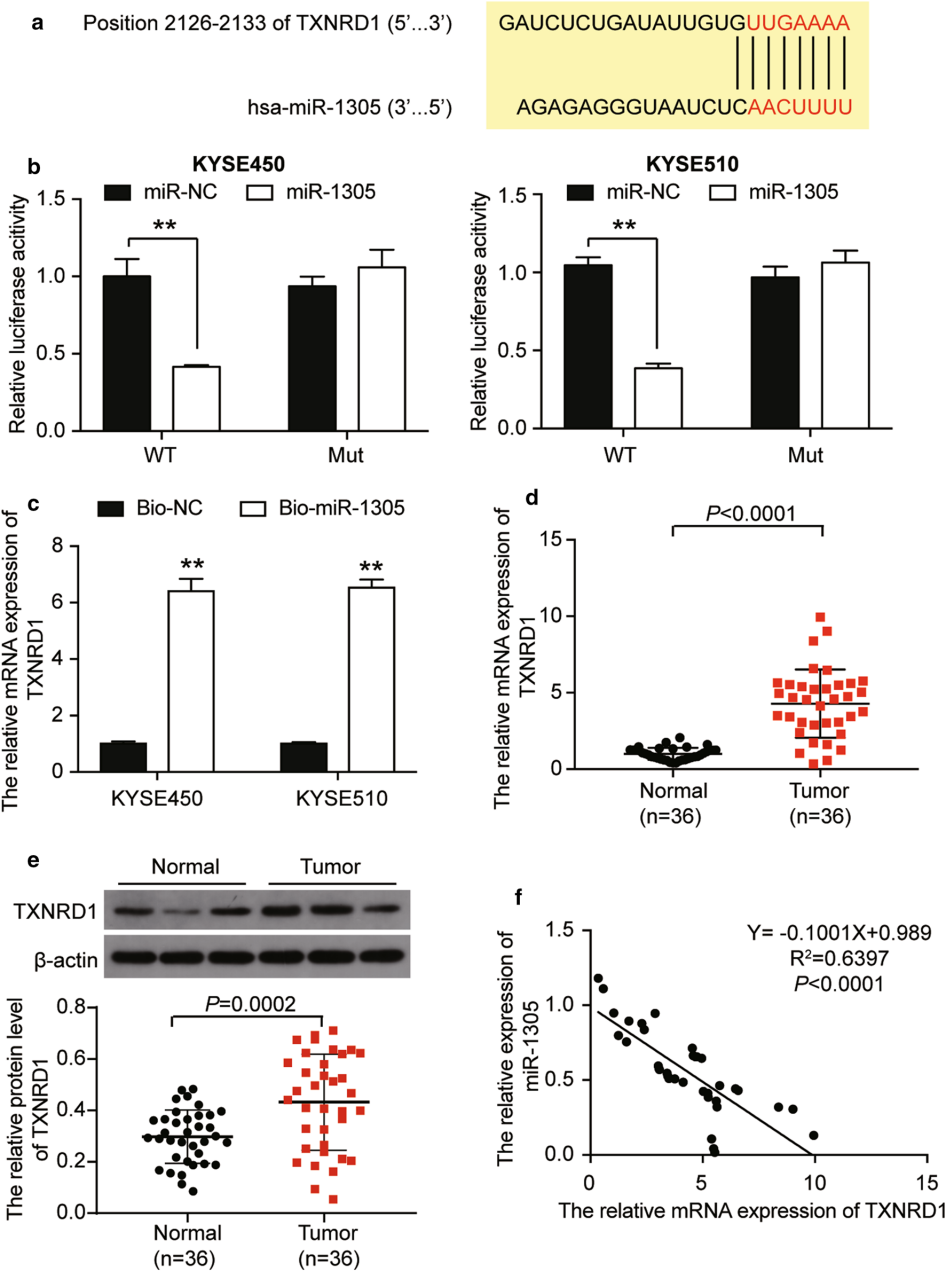
TXNRD1 was a target gene of miR-1305. **a** The binding sequence between TXNRD1 3′UTR and miR-1305 was predicted by TargetScan 7.0. **b** Luciferase reporter assay was used to observe the relationship between miR-1305 and TXNRD1. **P < 0.001, compared with miR-NC. miR-NC: miR-1305 negative control, WT: TXNRD1 wild type, Mut: TXNRD1 mutant type. **c** RNA pull-down assay was used to determine the relationship between miR-1305 and TXNRD1. **P < 0.001, compared with Bio-NC. Bio-NC: Bio-miR-1305 negative control. Bio-miR-1305: miR-1305 mimic-biotin. **d** RT-qPCR analysis showed that the expression of TXNRD1 was increased in ESCC tissues. **e** Pearson’s correlation analysis revealed that MiR-1305 had a negative relationship with TXNRD1. Each cellular experiment was independently repeated three times, and the data collected were displayed in the format of mean ± standard deviation (SD)

As shown in Fig. 1j, miR-1305 could not only interact with circ0120816 but also target TXNRD1. The TargetScan was used to predict the possible binding sites of miR-1305 in the 3′UTR of TXNRD1 (Fig. 6a). The results of the Luciferase reporter assay demonstrated that the co-transfection of miR-1305 mimic decreased the luciferase activity in ESCC cells transfected with wild type TXNRD1 by about 60% compared with the miR-NC group. However, no effect was found in ESCC cells co-transfected with mutant TXNRD1 and miR-1305 mimic (Fig. 6b). The RNA pull-down method was also employed to verify the relationship between miR-1305 and TXNRD1. Findings revealed that TXNRD1 expression in Bio-miR-1305 was 6 times as high as Bio-NC in ESCC cells. This outcome confirmed the direct interaction of miR-1305 with TXNRD1 in ESCC cells (Fig. 6c). RT-qPCR and western blot assay was later used to measure the mRNA and protein expression of TXNRD1 in ESCC tissues. The results revealed that the mRNA and protein level of TXNRD1 was dramatically upregulated in tumor tissues compared to adjacent healthy tissues (Fig. 6d, e). Besides, TXNRD1 expression had a negative association with miR-1305 expression (Fig. 6f). On the whole, these data revealed that TXNRD1 was a target gene of miR-1305 in ESCC development.

### MiR-1305 suppressed ESCC progression by targeting TXNRD1

**Fig. 7:**
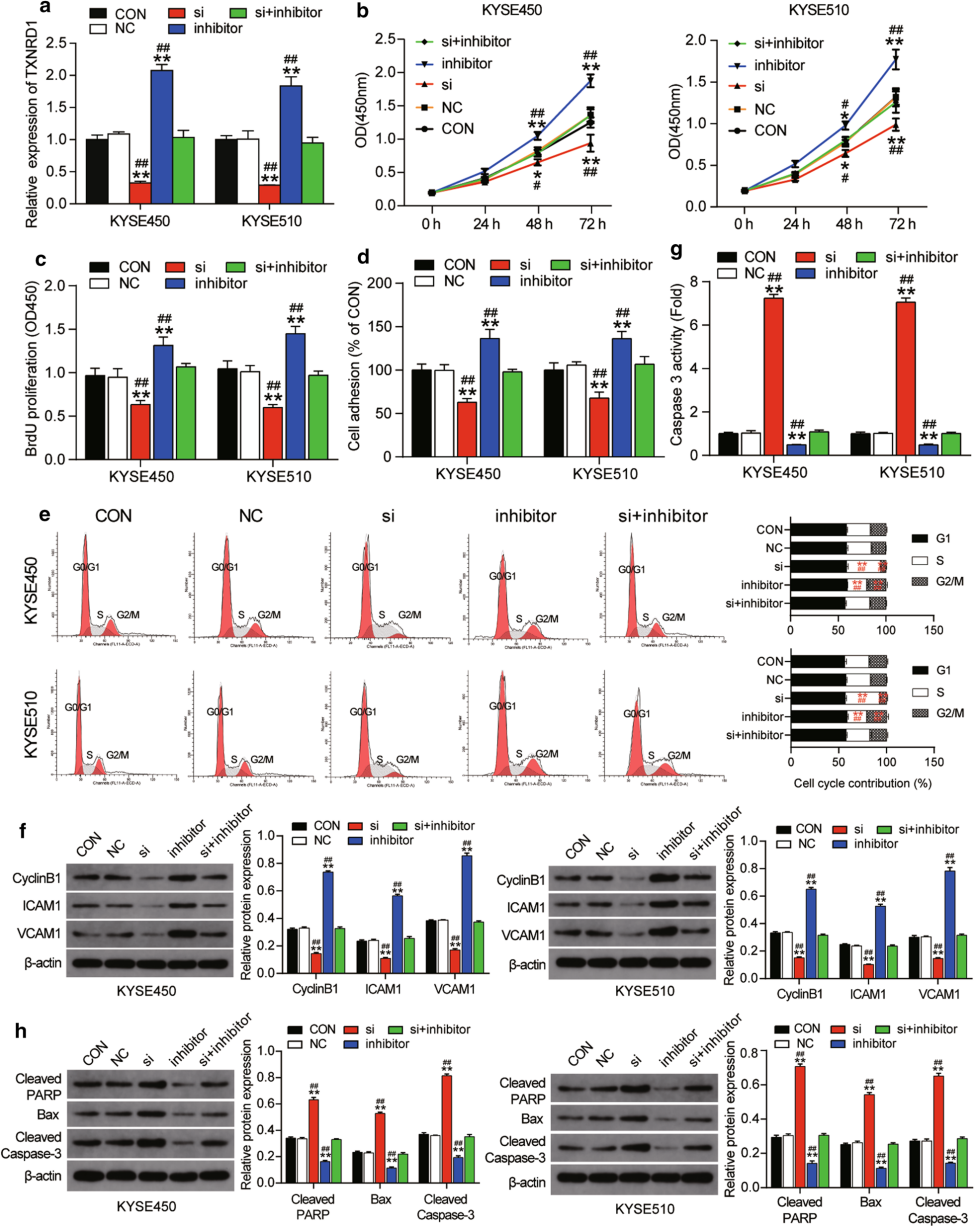
MiR-1305 suppressed ESCC progression by targeting TXNRD1. **a** RT-qPCR analysis of the expression of TXNRD1 after transfecting with TXNRD1 siRNA and/or miR-1305 inhibitor. **b** CCK-8 assay was performed to evaluate the cell viability of ESCC cells after transfection. **c** BrdU assay was conducted to evaluate the proliferation of ESCC cell lines after transfection. **d** Cell adhesion assay was used to assess the adhesion ability of ESCC cell lines after transfection. **e** Cell cycle assay was applied to evaluate the cell cycle in ESCC cell lines after transfecting with a flow cytometry system. **f** The expression of cyclinB1, ICAM1 and VCAM1 was detected in ESCC cell lines after transfecting with western blot assay. **g** Caspase 3 activity assay indicates the cell apoptosis of ESCC cell lines after transfection. **h** The expression of Cleaved PARP, Bax and Cleaved Caspase-3 was detected in ESCC cell lines after transfecting with western blot assay. *P < 0.05, **P < 0.001, compared with the blank control group. #P < 0.05, ##P < 0.001, compared with the co-transfection group of si-TXNRD1 plus miR-1305 inhibitor. CON: blank control, NC: negative control, inhibitor: miR-1305 inhibitor, si: TXNRD1 siRNA, si + inhibitor: TXNRD1 siRNA plus miR-1305 inhibitor. Each experiment was independently repeated three times, and the data collected were displayed in the format of mean ± standard deviation (SD)

To explore whether miR-1305 facilitated cell viability, proliferation and adhesion while inhibiting the apoptosis of ESCC cells by targeting TXNRD1, we transfected si-TXNRD1, miR-1305 inhibitor or si-TXNRD1 + miR-1305 inhibitor in KYSE450 and KYSE510 cells. RT-qPCR was first used to observe the expression of TXNRD1 in these groups. We observed about 60% down-regulation of TXNRD1 expression in the si-TXNRD1 group while the TXNRD1 expression level was twice as high as the blank control group in the miR-1305 inhibitor group (Fig. 7a). What’s more, the change in the TXNRD1 expression level in si-TXNRD1 and miR-1305 group was reversed by co-transfecting si-TXNRD1 and miR-1305 inhibitor (Fig. 7a). CCK-8 assay results later confirmed that si-TXNRD1 could inhibit cell viability, an outcome that was opposite to the positive effect of miR-1305 inhibitor (Fig. 7b). The co-transfection of si-TXNRD1 and miR-1305 inhibitor could attenuate the positive effect of miR-1305 inhibitor (Fig. 7b). Furthermore, BrdU assay results demonstrated that si-TXNRD1 restricted cell proliferation by 40% and that miR-1305 inhibitor enhanced it by about 35% compared to the blank control group in KYSE450 cells. A similar trend was also observed in KYSE510 cells (Fig. 7c). Moreover, the promotive effect of miR-1305 inhibitor in ESCC cell proliferation could be maintained with the miR-1305 inhibitor (Fig. 7c). Cell adhesion assay was further leveraged to explore the effect of TXNRD1 on ESCC cells. The result confirmed that si-TXNRD1 repressed cell adhesion by 40% and that miR-1305 inhibitor enhanced it by 40% compared to the blank control group in KYSE450 and KYSE510 cells. These changes were all compromised by the co-transfection of si-TXNRD1 and miR-1305 inhibitor (Fig. 7d). Similar to the BrdU assay results, the cell cycle assay results indicated that si-TXNRD1 caused the S phase arrest in ESCC cells. Moreover, in KYSE450 and KYSE510 cells co-transfected with si-TXNRD1 and miR-1305 inhibitor, the cell cycle was identical to that of the blank control group (Fig. 7e). Corresponding to the cell adhesion assay and cell cycle assay, the level of the cell cycle-related protein CyclinB1 and cell adhesion-related protein ICAM1 and VCAM1 were significantly downregulated in the si-TXNRD1 group compared to the control group. Moreover, the changed protein expression in the miR-1305 inhibitor group could be completely reversed by co-transfecting si-TXNRD1 (Fig. 7f). Caspase 3 activity assay results also demonstrated that si-TXNRD1 accelerated cell apoptosis to a seven-fold level, whereas the blank control group and miR-1305 inhibitor repressed it by 50% in ESCC cells. These effects were reversed by the co-transfection of si-TXNRD1 and miR-1305 inhibitor (Fig. 7g). The western blot data demonstrated that the levels of pro-apoptotic proteins (i.e., Cleaved PARP, Bax and Cleaved Caspase-3) in the si-TXNRD1 group were dramatically upregulated in contrast to the control group. Besides, the downregulation of the three pro-apoptotic proteins caused by miR-1305 inhibitor could be completely reversed by si-TXNRD1 (Fig. 7h). In short, the overall results confirmed that miR-1305 could promote cell viability, proliferation and adhesion and inhibit the apoptosis of ESCC cells in vitro by suppressing TXNRD1.

## Discussion

Reliable treatments for ESCC include surgical operations, radiotherapy and chemotherapy. Although these ESCC treatments have been improved significantly in the last 10 years, ESCC prognosis is still poor due to lack of early diagnosis and due to its diffuse and invasive nature. This research confirmed the existence of circ0120816 upregulation in ESCC and explored its specific effect on ESCC development. Our results indicated that circ0120816 facilitated ESCC progression via the miR-1305/ TXNRD1 axis.

Emerging circRNAs have been discovered to play an irreplaceable role in the progression of ESCC [66–69]. A research work uncovered that circ-TTC17 was apparently upregulated in ESCC cells, meaning it enhanced cell proliferation, migration and invasion [68]. In another research, researchers discovered a decrease in circ-SMAD7 in ESCC patients’ plasma could block tumor proliferation and migration [69]. Our experiment identified circ0120816, a novel circRNA that regulates ESCC progression. The circ0120816 acted as a tumor promoter: it strengthened cell viability, proliferation and adhesion, and inhibited cell apoptosis. Our in vivo study further confirmed that silencing circ0120816 attenuated the tumor growth and lung metastasis of ESCC. Several studies have also confirmed that circRNAs can influence protein-coding gene expression by competitive sponging for miRNAs. For instance, a study conducted by Rui-chao Li (2018) demonstrated that ciRS-7 overexpression extinguished the tumor-suppressive effects of miR-7 in facilitating ESCC malignant progression [67]. Consistent with this biological mechanism, circ0120816 was observed in this study to accelerate ESCC progression by sponging miR-1305. Based on these results, it can be concluded that circ0120816 could facilitate cell viability, proliferation, adhesion, and metastasis while suppressing cell apoptosis in regulating ESCC pathogenesis. Furthermore, we unveiled the high sensitivity and specificity level of circ0120816 in ESCC diagnosis and demonstrated its clinical diagnostic value for ESCC.

In 2015, miR-1305 was first discovered by Ng et al*.* to damage the periodontal ligament-derived stem cells of people who are prone to smoking [70]. Another study reported that a decrease in miR-1305 not only accelerated the metastasis of tumors but also aggravated the poor prognosis of NSCLC patients by inhibiting the expression of MDM2 [53]. In one research report, it was illustrated that a reduction of miR-1305 in triple-negative breast cancer could enhance the expression of RUNX2 and facilitate cancer aggressiveness [54]. Moreover, miR-1305 was found to restrict the activation of the AKT signaling pathway by competitively binding UBE2T in order to suppress the tumorigenicity of hepatocellular carcinoma cells [55]. All these results mentioned above consistently revealed that miR-1305 could block carcinogenesis. In our study, we also determined the repressive role of miR-1305 in ESCC progression by suppressing cell viability, proliferation and adhesion and facilitating cell apoptosis. To be more specific, we determined TXNRD1 as a downstream target gene of miR-1305 in the suppression of ESCC. It was found that miR-1305 acted as an inhibitory regulator in ESCC cells, thus decreasing the expression of TXNRD1.

TXNRD1 is a key enzyme that participates in the detoxification of reactive oxygen species (ROS) and redox signaling [71]. Interestingly, ROS has been discovered in various cancers. It has been shown to activate tumor signals and facilitate cell proliferation [72]. The corresponding reaction is that cancer cells will accelerate the levels of antioxidant proteins (such as TXNRD1), which can detoxify ROS to maintain redox balance and anti-apoptosis [72]. As described in some hepatocellular carcinoma cases, TXNRD1 was elevated to control the ROS level and maintain the tumorigenesis [73, 74]. These previous studies suggested that reduced TXNRD1 strengthened anticancer treatments. This suggestion was consistent with our research results. More specifically, TXNRD1 exhibited a higher expression in ESCC tissues and exerted a promotional effect on ESCC formation.

Furthermore, we presented compelling evidence that the post-transcriptional regulation of TXNRD1 was partly controlled by circ0120816. We also revealed that TXNRD1 could positively regulate cylclinB1 and adhesion molecule ICAM1 and VCAM1 to promote the proliferation and adhesion abilities of ESCC cells and negatively regulate pro-apoptotic proteins Cleaved PARP, Bax and Cleaved Caspase-3. It could also limit ESCC cell apoptosis, which was partially regulated by circ0120816 sponging miR-1305.

This experiment is not immune to several limitations despite our insightful findings. For instance, we did not design the experiment to explore the changes of ROS in ESCC cells. Such changes affect the level of cell proliferation and apoptosis and thus play important roles in tumorigenesis [72, 73, 75, 76]. Given that TXNRD1 can control ESCC progression by regulating ROS production, it is necessary to perform experiments that consider the effect of silencing TXNRD1 in ROS production. Future research should also explore the impact of ROS treatments on the cell apoptosis of ESCC cells. Apart from that, in the future, experiments should be performed to investigate the signaling pathways used by TXNRD1 to regulate cell cycle, cell adhesion and cell apoptosis expressions.

## Conclusions

**Fig. 8:**
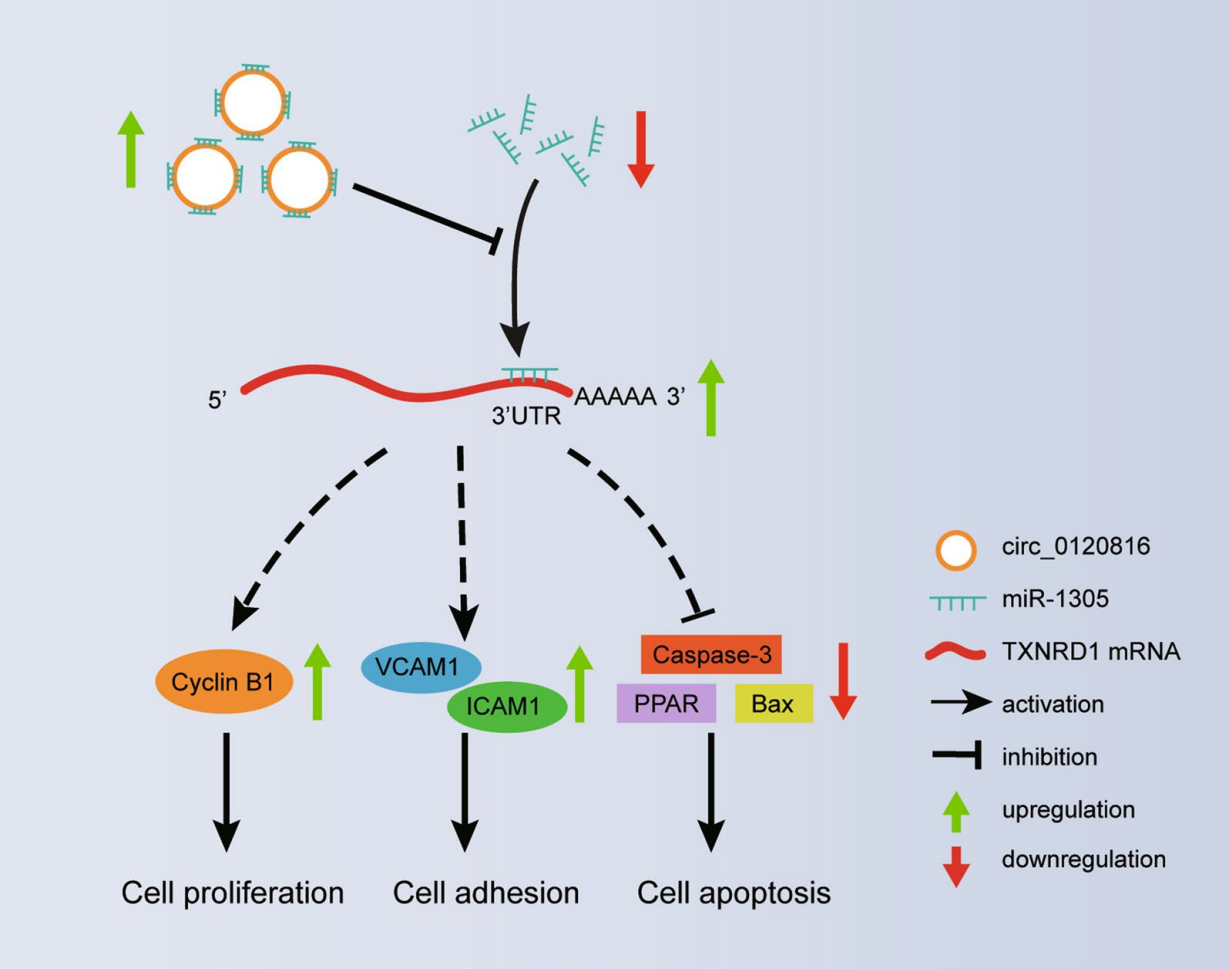
The schematic diagram of circ0120816/miR-1305/TXBRD1 axis working in ESCC tumorigenesis

In sum, our data offer more clarity on the mechanism used by circ0120816 to stimulate ESCC development. More specifically, we found that circ0120816 could facilitate ESCC growth by targeting miR-1305 to increase the expression of TXNRD1. We even discovered that circ0120816 not only promoted cell cycle-related proteins and adhesion molecules but also inhibited pro-apoptotic protein expression from aggravating ESCC development and progression (Fig. 8). Our findings also revealed that the circ0120816/miR-1305/TXBRD1 axis was activated in ESCC. We believe that this knowledge could aid in the improvement of ESCC treatments.

## Data Availability

The data used and analyzed during the current study are available from the corresponding author on reasonable request.

## Abbreviations

ESCC: Esophageal squamous cell carcinoma
circRNAs: Circular RNAs
miRNA: MicroRNA
TXNRD1: Thioredoxin reductase 1
Trx: Thioredoxin
RIP assay: RNA immunoprecipitation assay
Ago2: Anti-Argonaute2
ROS: Reactive oxygen species
